# Who are the intruders in medical education?

**DOI:** 10.1111/medu.15771

**Published:** 2025-07-05

**Authors:** Marco Antonio de Carvalho Filho, Megan Milota, Frederic William Hafferty, Ligia Cayres Ribeiro

**Affiliations:** ^1^ Wenckebach Institute, Research Program LEARN (Lifelong Learning Education and Assessment Research Network), University Medical Center Groningen University of Groningen Groningen The Netherlands; ^2^ Julius Center for Health Sciences and Primary Care University Medical Center Utrecht Utrecht The Netherlands; ^3^ Center for Professionalism and the Future of Medicine, Accreditation Council for Graduate Medical Education Chicago Illinois USA

## Abstract

Detailing how the hidden curriculum in medicine is full of social rituals and codes that mirror elite social structures, the authors outline how the intruder paradox can invite broader societal critique.

We felt deeply intrigued and inspired by the manuscript that introduces the concept of the *intruder paradox* as a critical counterpoint to the widely discussed *impostor phenomenon*.^1^ One of the most compelling contributions of this new concept is that it shifts the locus of the ‘problem’ from the individual psyche (impostor phenomenon) to the social structure within which this psyche is alleged to exist and communicate with both self and others (intruder paradox). Where the impostor phenomenon frames the experience of not belonging as a mistakenly internalised sense of fraudulence, the intruder paradox reframes it as an externally imposed experience of exclusion—especially for those who are seen to deviate from the historically dominant norms of medicine. In doing so, the concept invites us to interrogate the social architecture of medicine itself. Although conceptualised by the authors around the dimension of gender, the intruder paradox concept may inform intersecting processes of *othering* in medical education.

*The intruder paradox concept may inform intersecting processes of* othering *in medical education*.

So, inspired by this reframing, we offer a provocation: Who, exactly, are the *intruders* in medical education and practice? And what are the cultural blueprints that ‘culturally clone’ certain kinds of professionals while simultaneously signalling other individuals or groups as outsiders?^2^ We hope this reflection encourages further research into the structural dimensions of non‐belonging and exclusion in the medical profession.

Our central argument is that modern medical education is designed—perhaps unintentionally but nevertheless, structurally—for the success of white, wealthy, subservient, men. This design is not neutral; it is encoded in the rituals, expectations and rhythms of medical training. Let us explore these four dimensions more deeply:

*Modern medical education is designed—perhaps unintentionally but nevertheless, structurally—for the success of white, wealthy, subservient, men*.

Medicine, as institutionalised in the West, is a colonial construct. It evolved in parallel with European expansionism, often replacing or suppressing traditional healing practices across the Global South. This coloniality is embedded in and enacted by modern medical professionalism, which continues to privilege Eurocentric values: objectivity, neutrality, emotional detachment and rationality.^3^ These culturally specific values often act as gatekeepers to belonging. Trainees from non‐European backgrounds may experience their identities and epistemologies as incompatible with the ‘whiteness’ of medicine—leading not to *impostorism* in the psychological sense, but to an enforced outsider status, as in the suggested intruder paradox.^4^, ^5^

*Trainees from non‐European backgrounds may experience their identities and epistemologies as incompatible with the ‘whiteness’ of medicine*.

For instance, I, the first author of this commentary, a great‐great‐child of a traditional healer, vividly remember the first time I was exposed to a conversation about medical professionalism in a North‐American conference. Several practices I understood as normal in my cultural context, such as crying during consultations, giving a ride or becoming friends with patients, using the word ‘love’ in the context of professional relationships, were deemed ‘unprofessional’. It took me years to feel empowered to critically reflect and act upon these norms because they are framed as universally relevant, as if there is only one *right* way of being a doctor: a North‐American, North‐European way. One of the barriers to challenge this dominant narrative on medical professionalism is the fact that the global academic knowledge debate still excludes global south epistemologies. Often, in English, we are intruders.

Around the world, medical training is an expensive enterprise. In market‐driven education systems, this disproportionately excludes those from working‐class or first‐generation backgrounds. First, the selection procedures for medical schools often rely on an idea of meritocracy that considers the best candidates the ones who have acquired large amounts of knowledge during high school, privileging students who had the financial and social conditions to afford complementary (often private) education.^6^ Students who had the financial and social security to focus on their studies without the burden of having to contribute to their family incomes. Second, medical schools, in general, demand for full time dedication forcing students to remain economically dependent on their families. This dependence is even worse in countries where higher education is expensive, where student debt is sky rocketing.^7^ Finally, medicine sub‐specialisation is increasing the time spent on training, and it is taking longer for young doctors to join the workforce as independent practitioners.

But the costs are not only financial. The hidden curriculum of medicine is full of social rituals and codes—formal dinners, networking events, language games—that mirror elite social structures.^7^ Trainees not familiar with these rituals, or who feel alienated by them, are marked as different. They are not simply ‘failing to assimilate’; they are being structurally excluded.

*The hidden curriculum of medicine is full of social rituals and codes—formal dinners, networking events, language games—that mirror elite social structures*.

The culture of medicine is profoundly hierarchical. From the outset, students are socialised into a system where conformity is rewarded and questioning authority is punished. This top‐down hierarchy produces a culture where being subservient is confused with being professional. Trainees who resist, speak up, or challenge norms are labelled ‘difficult’, ‘not a good fit’ and thus sanctioned as ‘unprofessional’. This is often described as *weaponised professionalism*—a system where the standards of ‘professional behaviour’ are applied selectively to maintain existing hierarchies and structures of power and privilege, punishing non‐conforming attitudes that challenge the status quo.^8^ The result is not just exclusion but delegitimisation. In addition, professionalism can also become nostalgic, as when senior professionals idealise the past, demonise the new generations and actively resist the incorporation of new values in medical culture.^9^ As a consequence, the medical community loses the opportunity to be renewed and refreshed by the *intruders*.

*The medical community loses the opportunity to be renewed and refreshed by the* intruders.

Despite the increasing number of women in medical schools around the globe, the structure of the medical career continues to assume the life trajectory of a man. The most intense training years coincide with a woman's reproductive years, often forcing on women an impossible choice between personal and professional aspirations. Residents who become pregnant are still seen as betraying the group, disrupting the team, or lacking commitment—whereas male peers are rarely asked to justify fatherhood.^10^ While not the sole determinant, cultural norms within Western medicine perversely contribute to channelling women into specialties perceived as less prestigious and thus less ‘worthy’. This career structure does not accommodate plural pathways to success, and women are penalised.^11^ This career demands sacrifice, but it is much more intense for a woman. Besides, medicine still reeks of old‐fashioned masculinity, with its competitive atmosphere, no pain‐no gain work ethos, aggressive communication styles and high prevalence of moral and sexual harassment permeating the career trajectory.

*Medicine still reeks of old‐fashioned masculinity*.

Taken together, these four dimensions illustrate our argument that the experience of not belonging is less an aberration or individual failing as it is a symptom of a system that was never designed with diversity in mind. The impostor phenomenon asks: *Why do I feel like I don't belong?* The intruder paradox responds: *Because the structure was not built for you*. Overestimating the impostor phenomenon as a default conclusion risks holding the individual responsible for a process that is often more structural than psychological. The intruder paradox, however, invites a broader societal critique—one that interrogates the conditions under which belonging is granted, and more importantly, denied.

*The intruder paradox, however, invites a broader societal critique—one that interrogates the conditions under which belonging is granted, and more importantly, denied*.

As we reflect on these four structural pillars—whiteness, wealth, subservience and masculinity—we recognise that their influence is not universal. They intersect and manifest differently across national, institutional and cultural contexts.^5^ But what they share is a power to invisibly govern who belongs and who is made to feel like an intruder. This is important not only for education but for healthcare in general. Adopting a student‐centred education, democratic and participative is the foundational of a person‐centred care committed to social justice.

We invite our readers to consider: Who are the intruders in your institution—and, in turn, who is missing? And what needs to change—in both instances—to ensure that their presence no longer feels paradoxical, but essential? We still have a long way to go in democratising medical education and practice. We all are responsible for this change.

## CONFLICT OF INTEREST STATEMENT

The authors have no conflict of interest to declare and contribute equally to the conceptualisation and writing of this commentary.

## Data Availability

Data sharing not applicable to this article as no datasets were generated or analysed during the current study.
